# HCB101: a novel potent ligand-trap Fc-fusion protein targeting the CD47-SIRPα pathway with high safety and preclinical efficacy for hematological and solid tumors

**DOI:** 10.1186/s13045-025-01742-x

**Published:** 2025-10-23

**Authors:** Jiin-Tarng Wang, Chi-Ling Tseng, Han-Fang Teng, Pan-Hsien Kuo, Yun-Chih Cheng, Yi-Jing Chen, Yi-Hsuan Lu, Chun-Chung Wang, Tsai-Kuei Shen, Hong-Fan Wang, Pei-Lun Tsai, Yu-Chen Wu, Chien-Hsin Ho, Wei-Tse Sun, Yen-Cheng Li, Yi-Hsuan Lee, Yu-Jiun Hung, Mingyi Chen, Zihai Li, Zong Sean Juo, Wenwu Zhai, Scott Shi -Kau Liu

**Affiliations:** 1 HanchorBio Inc, No. 1, Sec. 1, Tiding Blvd. Neihu Dist, Taipei City, 114066 Taiwan; 2 Department of Pathology, UT Southwestern Medical Center, 2330 Inwood Rd, Dallas, 75390 TX USA; 3 Pelotonia Institute for Immuno-Oncology, The Ohio State University Comprehensive Cancer Center - James, 460 W 10th Avenue, Columbus, 43210 OH USA

**Keywords:** CD47, SIRP-alpha, HCB101, Macrophage, Phagocytosis, Cancer immunotherapy.

## Abstract

Cluster of differentiation 47 (CD47) delivers an inhibitory signal that suppresses phagocytosis and prevents immune clearance of tumor cells by interacting with signal regulatory protein alpha (SIRPα) on myeloid cells. Although blockade of the CD47-SIRPα axis is a promising immunotherapeutic strategy, clinical development has been hindered by on-target toxicities (e.g., severe anemia) and insufficient potency. Herein we report a third generation CD47-SIRPα inhibitor HCB101, a rationally designed SIRPα-Fc fusion protein generated from a large-scale screening of a structure-guided SIRPα extracellular domain (ECD) mutant library and fused to a human IgG4 Fc. HCB101 demonstrates high-affinity binding to CD47, robustly promotes macrophage-mediated phagocytosis of tumor cells without affecting red blood cells and exhibits unique advantages over current CD47-targeting agents, including Hu5F9-G4, TTI-622, and ALX148. In multiple xenograft cancer models, HCB101 induced significant inhibition of tumor growth as a single agent and showed synergistic anti-tumor effects when combined with anti-HER2 or anti-EGFR monoclonal antibodies. Additionally, HCB101 treatment increased the M1/M2 macrophage ratio in the tumor microenvironment, suggesting repolarization of tumor-associated macrophages (TAMs) toward a pro-inflammatory phenotype. No dose-limiting toxicities or hematologic adverse effects were observed in murine or non-human primate studies.

**Supplementary Information:**

The online version contains supplementary material available at 10.1186/s13045-025-01742-x.

## To the editor

**Fig. 1:**
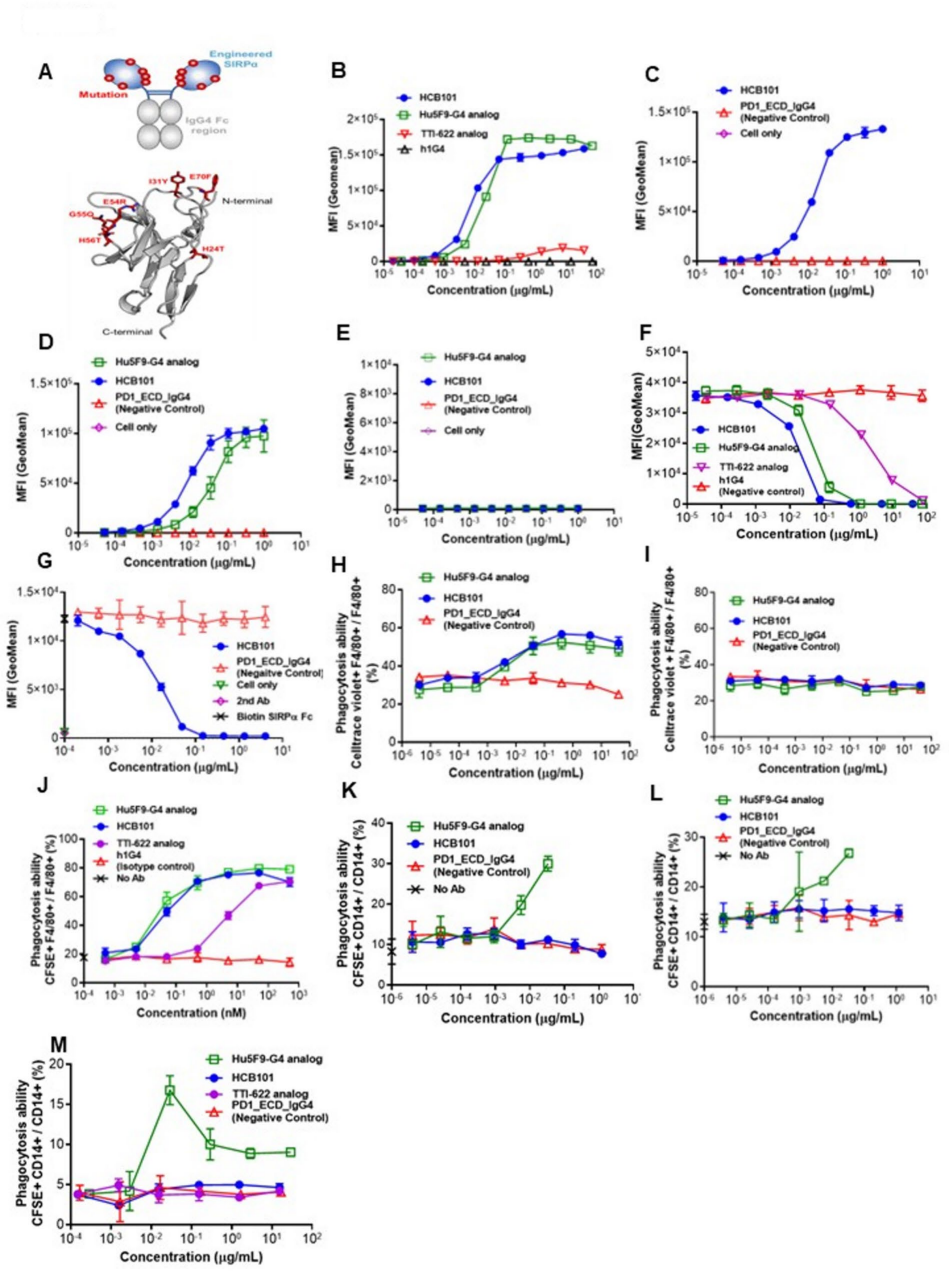
HCB101 schematic structure and in vitro activities. Structure of HCB101 is elucidated in (**A**). Top shows a schematic structure of HCB101, and the bottom is the simulated 3D structure of HCB101 which was visualized using PyMOL software for structural analysis, and the mutant sites on SIRPα ECD were labelled. HCB101 preferentially binds with high affinity to CD47-expressing cancer cells, but not to red blood cells. Serially diluted test articles were incubated with CD47-expressing Raji (**B**), FaDu (**C**), NCI-H82 (**D**), and hCD47 KO NCI-H82 (**E**) cells at 4 °C for 30 min. The binding was detected by PE-conjugated goat anti-human IgG Fcγ. Hu5F9-G4 and/or TTI-622 analogs are used as comparators. A PD1_ECD_IgG4 was used as negative control. HCB101-mediated blocking of human CD47 with SIRPα was assessed by flow cytometry (**F**-**G**): Serially diluted HCB101 or control molecules were incubated simultaneously with Raji (**F**) or FaDu (**G**) in the presence of the biotin-labeled human SIRPα-Fc. Bound ligands were detected by streptavidin-PE and analyzed by flow cytometry. The Hu5F9-G4 and TTI-622 analogs were used as comparators. PD1_ECD_IgG4 was used as a negative control; HCB101-induced macrophage-mediated phagocytosis of CD47-expressing NCI-H82 cells and human red blood cells were accessed (**H**–**M**): test articles were incubated simultaneously with macrophage cells (RAW264.7) and Celltrace™ Violet-labeled target cells, NCI-H82 (**H**) or CD47 KO NCI-H82 (**I**) or Raji (**J**) or RBCs isolated from three human donors (**K**-**M**) for two hours. The phagocytic activity was calculated as the percentage of CellTrace™ Violet^+^ F4/80^+^ cells within F4/80^+^ macrophages. The Hu5F9-G4 and TTI-622 analogs were used as comparators. PD1_ECD_IgG4 were used as negative controls, respectively. All data points are shown as mean ± SD for the triplicate determinations

CD47 is expressed on normal cells functioning as a “don’t eat me” signal by binding SIRPα on macrophages, thereby inhibiting phagocytosis [1, 2] (Fig.S1A). Tumor cells frequently overexpress CD47 to evade clearance by the innate immune system [3, 4]. Targeting the CD47-SIRPα pathway has therefore emerged as a promising immunotherapy in restoring macrophage-mediated phagocytosis [5, 6]. Several therapeutic modalities are currently under development [7, 8]. Challenges such as on-target anemia and limited efficacy hindered the success of these agents [6]. To obtain a superior efficacy with lower toxicities, we developed a third generation CD47-SIPRα inhibitor, HCB101, that is a rationally engineered SIPRα protein incorporating six targeted mutations in the ECD domain followed by fusion to the human IgG4-Fc (Fig. 1A).

HCB101 shows high binding to human and cynomolgus CD47_ECD (Fig.S1B and Table S1−3) and to human CD47-expressing cells (Fig. 1B-C). It exhibits a dose-dependent binding to NCI-H82 cells but not to human CD47 KO NCI-H82 cells, indicating that it binds specifically to NCI-H82 through CD47 (Fig. 1D-E). HCB101 blocks binding of SIRPα-Fc to CD47-expressing tumor cells (Fig. 1F-G, Fig.S4), more efficiently than Hu5F9-G4 [9] and TTI-622 analogs [10]. HCB101-mediated phagocytosis was approximately 100-fold higher than that of TTI-622 and is comparable to that of Hu5F9-G4 (Fig. 1H-J). HCB101 binds to erythrocytes with lower affinity and does not trigger significant phagocytosis of RBCs in-vitro (Fig.S2A-C). Unlike Hu5F9-G4, HCB101 did not induce the phagocytosis of freshly collected human erythrocytes, suggesting that HCB101 has a potentially better safety profile (Fig. 1K-M).

**Fig. 2:**
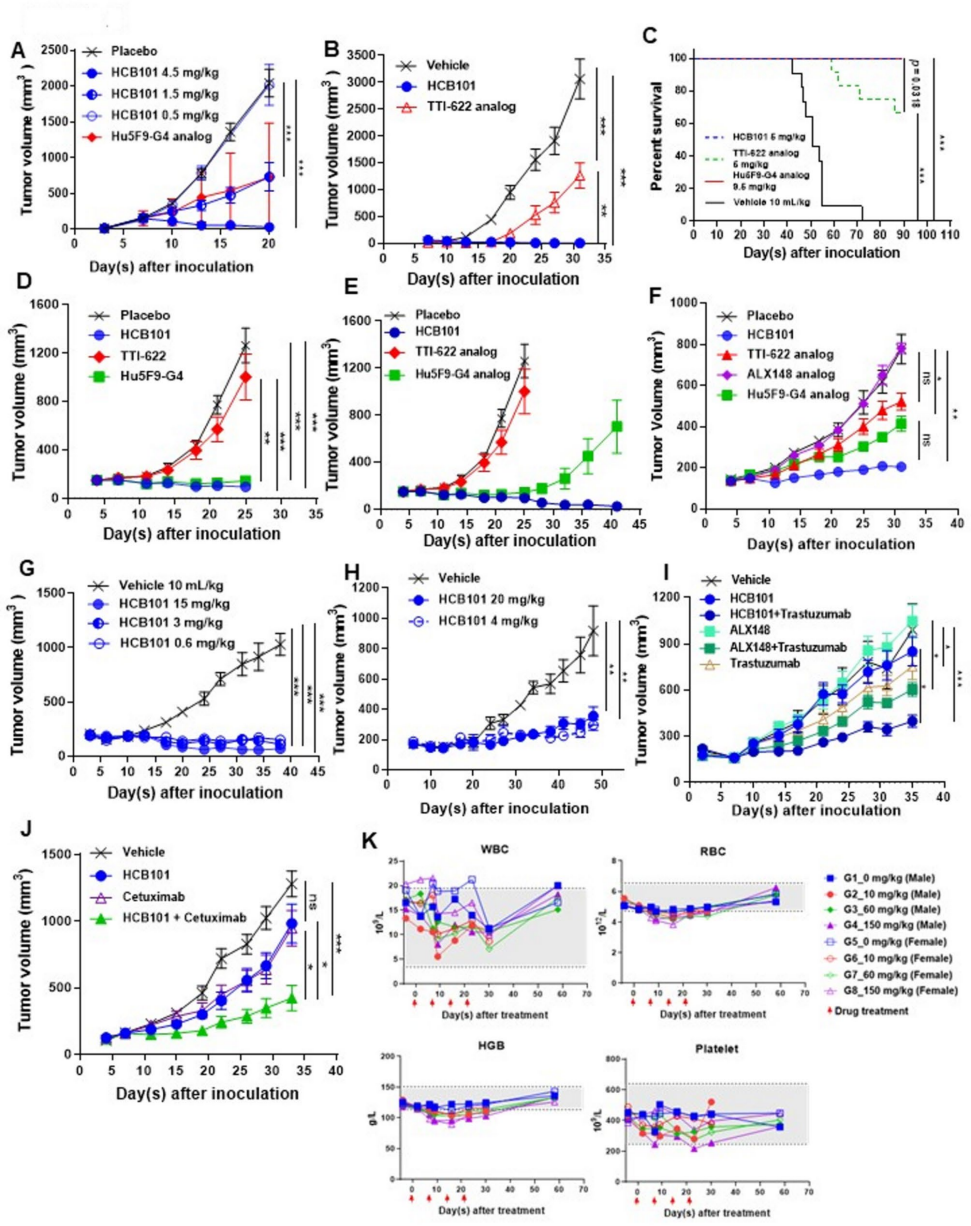
Anti-tumor activity of HCB101 in various xenograft models. (**A**) NOD/SCID mice were engrafted with Raji cells and treated with HCB101 at doses of 4.5, 1.5, and 0.5 mg/kg once weekly for two weeks (*n* = 10 per group). (**B**) NOD/SCID mice were intraperitoneally injected with HCB101 or TTI-622 analog five times per week for four weeks, beginning on day3 following Daudi tumor inoculation (*n* = 8 per group). (**C**) KG-1a was intravenously administrated into NPG™ mice (*n* = 12 per group). Mice were treated with HCB101 or TTI-622 analog five times per week for two weeks. The mouse survival was monitored daily. (**D** & **E**) NCI-H82 xenograft-bearing NOD/SCID mice were treated with HCB101, TTI-622 analog, or Hu5F9-G4 analog on the indicated days (*n* = 10 per group). (**F**) NOD/SCID mice were inoculated with WiDr cells and then treated with HCB101, TTI-622 analog, ALX148 analog, or Hu5F9-G4 analog when tumor volumes reached 100–200 mm^3^. Treatments were administered twice weekly for 3.5 weeks (*n* = 8 per group). (**G**) MDA-MB-453 cells were inoculated into NOD/SCID mice. Upon reaching a tumor volume of 100–200 mm^3^, mice received intraperitoneal injections of HCB101 at various doses twice weekly for three weeks and once weekly for an additional week (*n* = 5 per group). (**H**) NOD/SCID mice engrafted with SNU-C1 cells were treated with HCB101 at doses of 20 and 4 mg/kg, administered twice weekly for four weeks (*n* = 5 per group). (**I**) In NCI-N87 gastric cancer-bearing mice, treatment of 3 mg/kg for HCB101 or ALX148, with or without trastuzumab at 3 mg/kg, was initiated when tumor size reached 100–200 mm^3^. Treatments were administered biweekly for four weeks (*n* = 8 per group). (**J**) NOD/SCID mice bearing SW48 xenografts were treated with 10 mg/kg of HCB101, cetuximab, or a combination of both agents twice weekly for four weeks (*n* = 8 per group). All tumor volume data are presented as mean ± SEM. (**K**) Hematology in cynomolgus monkeys. WBC, RBC, HGB, and platelet counts in monkeys treated weekly with HCB101 (10–150 mg/kg) or placebo for 4 weeks. Data are mean ± SEM

HCB101 shows excellent anti-tumor activities across all tested in vivo xenograft cancer models. In Raji, TGI of HCB101 was 99%, 65%, and 1% at dosing of 4.5, 1.5 and 0.5 mg/kg respectively (Fig. 2A, Fig.S5A). In Daudi, TGI of HCB101 and TTI-622 was 102% and 59% respectively at dosing of 10 mg/kg, indicating HCB101 had better in-vivo efficacy (Fig. 2B). In KG-1a-bearing mice, treatment separately with HCB101 and Hu5F9 achieved a 100% survival rate while only 66.7% was observed in TTI-622 group (Fig. 2C). In NCI-H82, TGI of HCB101 and Hu5F9-G4 groups was 105% and 101% respectively, compared to 23% for TTI-622 group on day 25 post-tumor inoculation (Fig. 2D). In HCB101 and Hu5F9 groups, two more treatments were given on day 28 and day 32. On day 41, the ATV of Hu5F9 group increased to approximately 700 mm^3^, while ATV of HCB101 group decreased to 0 mm^3^ for all mice, suggesting HCB101 has much better efficacy as monotherapy for NCI-H82 cells (Fig. 2E). In WiDr bearing NOD/SCID mice, TGI of HCB101, TTI-622, ALX148 [11] and Hu5F9-G4 were 89%, 39%, −1% and 56% respectively, suggesting that HCB101 had superior anti-tumor efficacy than the analogs (Fig. 2F, Fig.S5B). In MDA-MB-453, HCB101 had potent anti-tumor activity (Fig. 2G) with the TGI of 108%, 100%, and 95% at the dose of 15, 3, and 0.6 mg/kg respectively. In SNU-C1, TGI of HCB101 at doses of 4 and 20 mg/kg was 76% and 84% respectively (Fig. 2H). A head-to-head study using NCI-N87 cells in NOD/SCID mice demonstrated that the combination of the HCB101 with trastuzumab had a superior tumor suppression compared to ALX148 with trastuzumab (Fig. 2I, Fig.S5C, Table S5). A similar study using SW48 cells in NOD/SCID mice, HCB101 combining with cetuximab results in improved anti-tumor efficacy (Fig. 2J). ADCP significantly increased in combination of HCB101 and trastuzumab (Fig.S7). The numbers of hematopoietic cells including the M1 macrophage were all significantly increased in tumors isolated from NOD/SCID mice treated with HCB101 or Hu5F9 but not in tumors treated with TTI-622 or ALX148 analogs (Fig.S3A-C). For the M2 macrophage, no significant increase was observed (Fig.S3D). The M1/M2 ratio was significantly increased within WiDr tumors treated with HCB101 (Fig.S3E). HCB101-induced activation of immune cells were also detected in the hCD47-CT26 syngeneic model (Fig.S6), indicating that HCB101 treatment resulted in a more favorable immune profile for cancer control. We also found that IgG effector function is essential for HCB101-mediated phagocytosis (Fig.S4A-E). A favorable safety and dose-proportional pharmacokinetics with corresponding target engagement were observed in non-human primates (Fig. 2K, Fig.S8).

In conclusion, HCB101’s design minimizes RBC binding and antigen sink, enabling safer, higher dosing. These findings position HCB101 as a differentiated, next-generation CD47-SIRPα inhibitor combining potent efficacy with a strong safety margin in solid tumor treatment—two historically opposing features in this drug class.

## Supplementary Information

Supplementary Material 1.

Supplementary Material 2.

Supplementary Material 3.

Supplementary Material 4.

Supplementary Material 5.

Supplementary Material 6.

Supplementary Material 7.

Supplementary Material 8.

Supplementary Material 9.

## Data Availability

No datasets were generated or analysed during the current study.

## Abbreviations

ADCP: Antibody dependent cytotoxic phagocytosis
ATV: Average tumor volume
CD47: Cluster of Differentiation 47
CDX: Cell line-derived xenograft
ECD: Extracellular domain
IgG1: Immunoglobulin G1
IgG4: Immunoglobulin G4
MDMs: Monocyte-derived macrophages
KO: Knock-out
NOD/SCID: Non-obese diabetic/severe combined immunodeficiency
RBCs: Red blood cells
SIRPα: Signal regulatory protein alpha
TAM: Tumor associate macrophage
TGI: Tumor growth inhibition
