# Supplementary material for: HCB101: a novel potent ligand-trap Fc-fusion protein targeting the CD47-SIRPα pathway with high safety and preclinical efficacy for hematological and solid tumors
Source: J Hematol Oncol. 2025 Oct 23;18:87. doi: 10.1186/s13045-025-01742-x (PMC12548202; doi:10.1186/s13045-025-01742-x)
Supplement: Supplementary file 1 — Supplementary Material 1. [file 13045_2025_1742_MOESM1_ESM.docx]

**Additional File 1**

1. **Supplemental material and methods:** Page 1-5
2. **Supplementary Tables:** Page 6-7
3. **Supplementary Figure Legends:** Page 7~11
4. **Materials and Methods**

**SIRPα Library Construction and Screening**

A combinatorial phage display library encoding amino acids 31–148 of the human SIRPα IgV domain (GenBank: CAA71403.1) was constructed using structure-guided mutagenesis targeting the BC (24-32 a.a.), C’D (54-56 a.a.), and DE loops (66-73 a.a.), which comprise the interaction interface with CD47. Mutated fragments were assembled via PCR and cloned into the pADL™-23c vector. The library, with an estimated diversity of 1.98 × 10⁸ variants, was transformed into *E. coli* TG1 cells by electroporation.

**Biopanning and Clone Selection**

Negative selection against human RBCs preceded four rounds of positive selection using CD47-expressing Raji cells, recombinant CD47-Fc, and HCT116 cells. Enriched clones were amplified and screened via ELISA and ligand-blocking assays. Fifty-six clones underwent further characterization, including whole-cell binding, thermostability, and RBC interaction. Fourteen lead clones were selected for IgG4-Fc fusion. Among the IgG4-Fc clones, we further select those with significantly reduced affinity for CD47 and phagocytosis on human RBCs, while retaining high-affinity binding and phagocytosis functional activity toward CD47 on tumor cells.

**Protein Expression and Purification**

SIRPα-Fc fusion constructs were cloned into pAS-puro and transiently expressed in CHO-S cells. Proteins were purified using Protein A chromatography and analyzed by HPLC-SEC.

**Cell Lines and Culture**

Human tumor cell lines (e.g., Raji, Daudi, NCI-H82, SW48, KG-1a, NCI-N87) and RAW264.7 were obtained from ATCC (American Type Culture Collection) or BCRC (Bioresource Collection and Research Center) and cultured under standard conditions. CD47 knockout NCI-H82 cells were generated using CRISPR/Cas9. Media were supplemented with FBS, glucose, and sodium pyruvate as appropriate.

**Animal Studies**

Female NOD/SCID and NPG™ mice (5–6 weeks old) were purchased from BioLASCO Taiwan and used for xenograft models. Tumor cells were implanted subcutaneously or intravenously. Treatment with HCB101 or controls was administered intraperitoneally. The doses used in different tumor models were based on: (1) preliminary dose-ranging studies showing optimal efficacy at these levels and (2) comparison to published effective doses of comparator agents (Hu5F9-G4, TTI-622, and ALX148). The drug doses and group sizes of each study were illustrated in the figure legend. The doses of HCB101 are equimolar to the comparators. Tumor volume and survival were monitored, and tumor growth inhibition rate (%TGI) was calculated. All procedures complied with institutional animal care guidelines.

**Binding and Functional Assays**

Binding of HCB101 to recombinant CD47 was assessed by ELISA and bio-layer interferometry (BLI). The binding of HCB101 to cell-surface CD47 on tumor cells was evaluated by incubating cells with serially diluted antibodies, followed by PE-conjugated anti-human IgG and analysis on a CytoFLEX cytometer (Beckman Coulter). Blocking of ligand binding was assessed by incubating CD47-expressing cells with HCB101 and biotin-labeled hSIRPα-Fc, detected via PE-streptavidin, with quantification by flow cytometry. Hemagglutination assays were performed using RBCs from healthy donors. Freshly isolated human RBCs were incubated overnight with serially diluted antibodies. Hemagglutination was visually scored the next day. Phagocytosis assays employed RAW264.7 or human monocyte-derived macrophages incubated with labeled target cells. Phagocytosis was measured by the percentage of macrophages that contained engulfed target cells, as determined by flow cytometry.

**Tumor Dissociation and TAM Analysis**

Tumors collected from mice were dissociated using enzymatic digestion and mechanical disruption. Single-cell suspensions were stained with viability dye and antibodies targeting CD45, CD11b, F4/80, CD80, and CD206. Flow cytometric analysis was performed to quantify the polarization of tumor-associated macrophages (TAMs).

**Non-Human Primate Toxicity**

**A GLP-compliant repeat-dose toxicity study in cynomolgus monkeys.** A 4-week repeat-dose toxicity study of HCB101 (10–150 mg/kg, i.v.) was conducted in cynomolgus monkeys, including a 4-week recovery phase to assess reversibility of any toxicity. Thirty-two monkeys were randomly assigned to three HCB101 dose groups and one placebo group. Monkeys in the high-dose group (150 mg/kg) included 5/sex, while low- and mid-dose groups (10 and 60 mg/kg) included 3/sex/group. All animals were dosed once weekly for 4 weeks. At study end, subsets of animals were euthanized for sample collection, while recovery animals were observed for an additional 26 days. Hematology parameters (WBC, RBC, HGB, and platelets) were measured during the dosing period.

**Pharmacokinetics and receptor occupancy.** Single-dose pharmacokinetics (PK) of HCB101 were assessed in cynomolgus monkeys at 3, 10, and 60 mg/kg. Serum drug concentrations were measured by ELISA at multiple time points from pre-dose to day 29. Receptor occupancy (RO) on CD3⁺ T cells was determined by flow cytometry using anti-human IgG4 Fc detection at corresponding time points. Median fluorescence intensity (MFI) was used to calculate %RO.

**Statistical Analysis**

*In vitro* data are presented as mean ± standard deviation (SD) to represent variability among technical replicates. *In vivo* results are shown as mean ± standard error of the mean (SEM) to reflect the precision of group means derived from independent biological replicates. Statistical significance was determined using Student’s *t*-test for comparisons between two groups or one-way ANOVA followed by Bonferroni's post hoc tests for multiple group comparisons. Significance thresholds were set at P<0.05 (*), P<0.01(**), p < 0.001 (***), and p < 0.0001 (****).

1. **Supplementary Tables**

**Supplementary Table S1.** The EC_50_ and EC_90_ (50% and 90% maximal effective concentration) of the CD47 binding affinity of HCB101 and Hu5F9-G4 analog

|  | **EC_50_ (ng/ml)** | **EC_90_ (ng/ml)** |
| --- | --- | --- |
| **HCB101** | 13.8 | 80.1 |
| **Hu5F9-G4 analog** | 47.29 | 271.14 |
| **PD1_ECD_IgG4**  **(Negative Control)** | N.D.* | N.D.* |

N.D.*: Not Detected

**Supplementary Table S2.** Binding kinetics of HCB101 to human CD47_ECD

| **Experiment No.** | **Human CD47_ECD** | | |
| --- | --- | --- | --- |
|  | **ka [1/(M s)]** | **kd [1/s]** | **Kd [M]** |
| Experiment 1 | 6.39 ± 0.11×10^5^ | 3.19 ± 0.09×10^-4^ | 4.99 ± 0.17×10^-9^ |
| Experiment 2 | 6.69 ± 0.44×10^5^ | 4.17 ± 0.24×10^-4^ | 6.24 ± 0.26×10^-9^ |
| **Average** | 6.54 ×10^5^ | 3.68×10^-4^ | 5.62×10^-9^ |

**Supplementary Table S3.** Binding kinetics of HCB101 to cynomolgus monkey CD47_ECD

| **Experiment No.** | **Cynomolgus CD47_ECD** | | |
| --- | --- | --- | --- |
|  | **ka [1/(M s)]** | **kd [1/s]** | **KD [M]** |
| Experiment 1 | 2.78 ± 0.10×10^5^ | 4.00 ± 0.08×10^-3^ | 14.4 ± 0.42×10^-9^ |
| Experiment 2 | 2.96 ± 0.16×10^5^ | 4.29 ± 0.25×10^-3^ | 14.6 ± 0.21×10^-9^ |
| **Average** | 2.87×10^5^ | 4.15×10^-3^ | 14.5×10^-9^ |

**Supplementary Table S4.** HCB101-mediated blocking of human CD47 binding to human SIRPα.

| **IC_50_ (ng/mL)** | **Raji** | **FaDu** |
| --- | --- | --- |
| HCB101 | 16.13 | 12.94 |
| Hu5F9-G4 analog | 48.4 | N.D.* |
| TTI-622 | 2360.0 | N.D.* |
| PD1_ECD_IgG4  (Negative Control) | N.D. | N.D. |

N.D.*: Not Detected

**Supplementary Table S5.** The %TGI value of HCB101 and ALX148 in combination with trastuzumab

| **Day 35 after incubation** | **Mean ± SEM** | **%TGI** | ***P* value¹** |
| --- | --- | --- | --- |
| HCB101 3 mg/kg | 851.8± 93.92 | 17% | > 0.9999 |
| HCB101 3 mg/kg+ Trastuzumab 3 mg/kg | 398±39.47 | 71% | 0.0021** |
| ALX148 3 mg/kg | 1047±108.1 | -7% | > 0.9999 |
| ALX148 3 mg/kg + Trastuzumab 3 mg/kg | 603.3±43.32 | 47% | 0.1374 |
| Trastuzumab 3 mg/kg | 753.2±87.74 | 29% | > 0.9999 |
| Vehicle 10 mL/kg | 990.1±168.7 |  |  |

¹ *P* values were calculated using one-way ANOVA followed by Bonferroni’s post hoc test.

1. **Supplementary Figure Legends**

**Figure S1. Schematic diagram of possible mechanism of action of HCB101 and CD47 binding affinity.** **(**A) Schematic diagram of possible mechanism of action of HCB101. (B) The binding affinity of serially diluted HCB101 or control molecules to human CD47_ECD proteins was determined by ELISA. Hu5F9-G4 analog was used as the positive control. PD1_ECD_IgG4 served as the negative control. All data points are shown as mean ± SD for the triplicate determinations.

**Figure S2.** **Binding activity of HCB101 to RBCs.** Serial diluted HCB101 and test articles were incubated with RBCs [^10^](#_ENREF_10) at 4°C for 30 min. PE-conjugated goat anti-human IgG Fcγ was used to detect the binding activity. Human RBCs bound with test articles were analyzed by flow cytometry. The Hu5F9-G4 analog was used as the comparator. The PD1_ECD_IgG4 or h1G4 served as the negative control. All data points are shown as mean ± SD for the triplicate determinations.

**Figure S3. Numeration of M1 and M2 macrophages isolated from WiDr tumors treated with HCB101 and other CD47 blocking agents.** (A~D) point chart demonstrated that the changes in the numbers of hematopoietic cells, total macrophage, M1 macrophage, and M2 macrophage after treatment of WiDr-bearing mice with HCB101, TTI-622, ALX148, and Hu5F9-G4 analogs (8 mice per group). (E) The ratio of M1 and M2 after treatment of WiDr-bearing mice with HCB101, TTI-622 analog, ALX148 analog, and Hu5F9-G4 analog (8 mice per group). All data points are shown as mean ± SEM for the triplicate determinations.

**Figure S4. IgG effector function is essential for HCB101-facilited phagocytosis.** (A) The binding affinity of HCB101 and other CD47 blocking agents to CD47 on Raji cells was assessed by flow cytometry. (B) The ability of HCB101 and other agents to inhibit ligand binding was evaluated using CD47-expressing Raji cells via FACS analysis. (C) Phagocytic activity was measured by co-culturing Raji cells (target) with RAW264.7 cells (effector) at a 1:1 ratio and quantified as the percentage of CellTrace™ Violet+ F4/80+ cells within F4/80+ macrophages. hIgG4 (anti-PD1 Ab) was used as the negative control. (D) *In vivo* anti-tumor activity was assessed in WiDr-bearing mice treated with HCB101, TTI-622 analog, ALX148 analog, and Hu5F9-G4 analog. (E) Phagocytosis activity was assayed in the presence of HCB101 and the HCB101_N197A mutant, where the N297A mutation refers to the substitution of asparagine (Asn, N) with alanine (Ala, A) at position 297 in the constant region (Fc domain) of the IgG heavy chain, according to the EU numbering scheme. hIgG4 was used as the negative control. All data points are expressed as the mean ± SEM from triplicate determinations.

**Figure S5. Representative images of NOD/SCID mice bearing Raji (A), WiDr (B), and NCI-N87 (C) tumors after indicated treatments.** Experimental procedures are detailed in the Materials and Methods section.

**Figure S6. Effect of HCB101 in the hCD47-CT26 syngeneic BALB/c model.** **(A)** Anti-tumor efficacy of HCB101. **(B)** Profiling tumor-infiltrating immune cells. BALB/c mice were implanted subcutaneously with 1 × 10⁶ hCD47-CT26 cells. When tumors reached ~50 mm³, mice were randomized (n = 6/group) and treated with HCB101 (20 mg/kg, i.p.) or vehicle twice weekly. Tumor volumes were monitored, and efficacy was expressed as tumor growth inhibition (TGI). At study end (vehicle, d29; HCB101, d34), tumors were dissociated and analyzed by flow cytometry for CD3⁺ T cells, CD8⁺ T cells, macrophages, conventional dendritic cells (cDCs), and myeloid-derived suppressor cells (MDSCs). Data are shown as mean ± SEM; tumor growth was analyzed by one-way ANOVA with Bonferroni’s post hoc test (**p < 0.01), and immune profiling by independent t-test (*p < 0.05).

**Figure S7.** **Effect of HCB101, trastuzumab, or their combination on RAW264.7 macrophage-mediated phagocytosis of NCI-N87 cells.** Phagocytic activity was evaluated by co-culturing NCI-N87 target cells with RAW264.7 effector cells at a 2:1 ratio for 2 h at 37 °C and quantified as the percentage of CellTrace™ CFSE⁺F4/80⁺ cells within the F4/80⁺ macrophage population. Human IgG4 isotype antibody was included as a negative control.

**Figure S8. Pharmacokinetic and receptor occupancy profiles of HCB101 in Cynomolgus Monkeys.**

**(A)** Single-dose PK of HCB101 in cynomolgus monkeys at 3, 10, or 60 mg/kg. Serum concentrations were measured by ELISA at indicated time points; data are shown as mean ± SD. **(B)** Receptor occupancy (RO) of HCB101 on CD3⁺ T cells in cynomolgus monkeys. The CD3^+^ T cells RO was assessed at the indicated time points using flow cytometry.
