# Supplementary figures and images for: HCB101: a novel potent ligand-trap Fc-fusion protein targeting the CD47-SIRPα pathway with high safety and preclinical efficacy for hematological and solid tumors
Source: J Hematol Oncol. 2025 Oct 23;18:87. doi: 10.1186/s13045-025-01742-x (PMC12548202; doi:10.1186/s13045-025-01742-x)

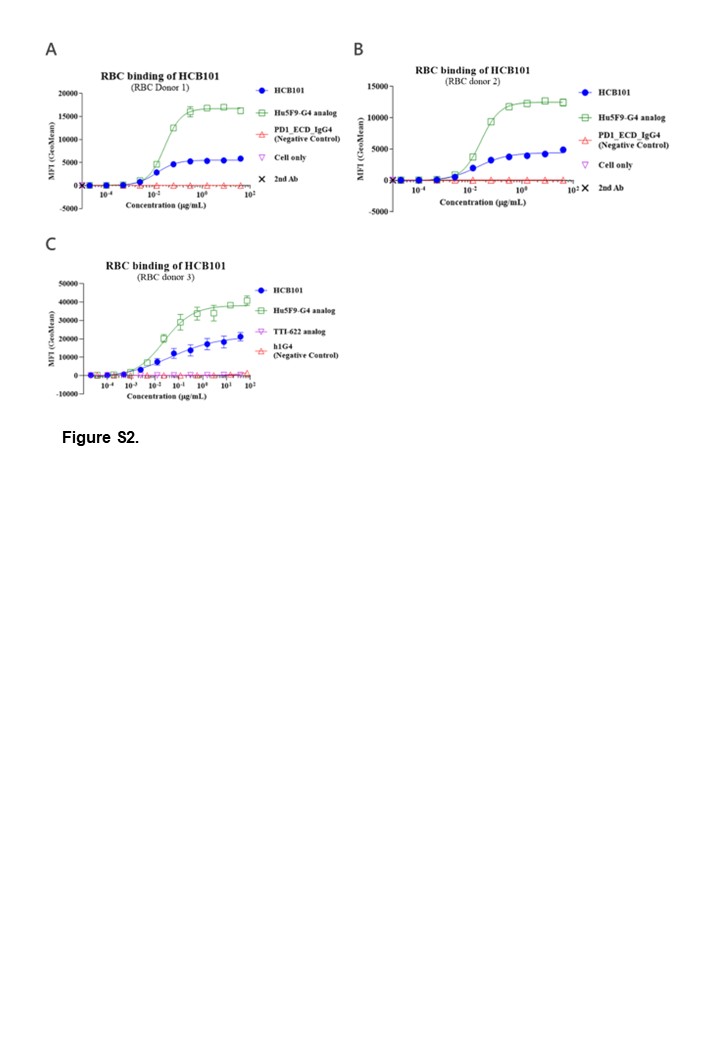

Supplement: Supplementary file 2 — Supplementary Material 2. [file 13045_2025_1742_MOESM2_ESM.jpg]

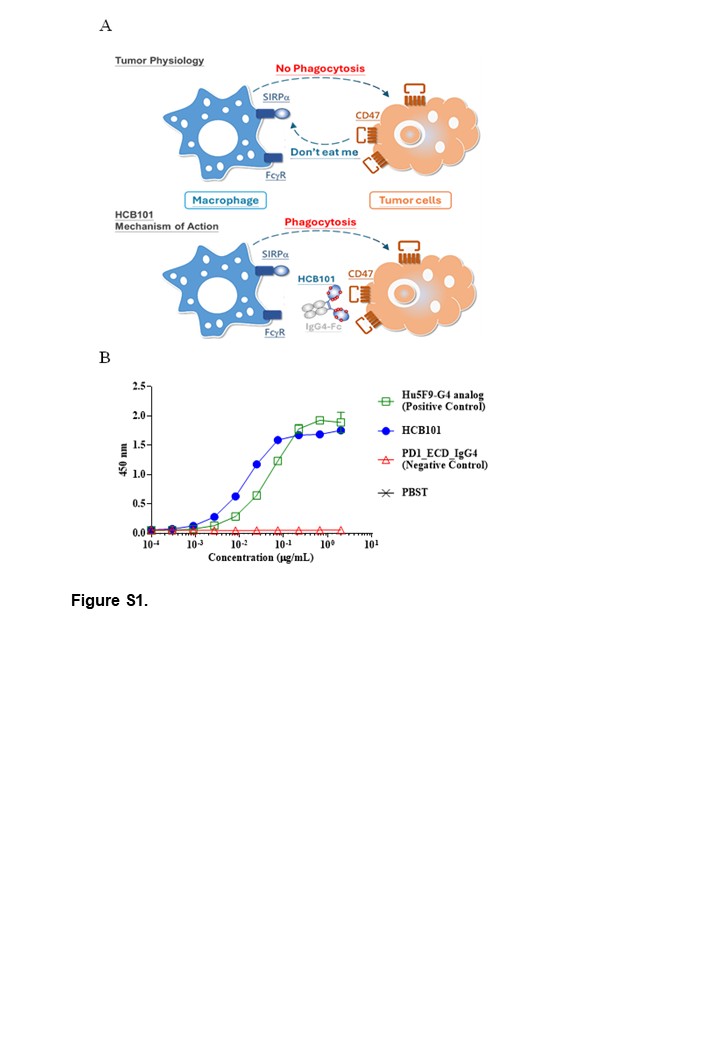

Supplement: Supplementary file 3 — Supplementary Material 3. [file 13045_2025_1742_MOESM3_ESM.jpg]

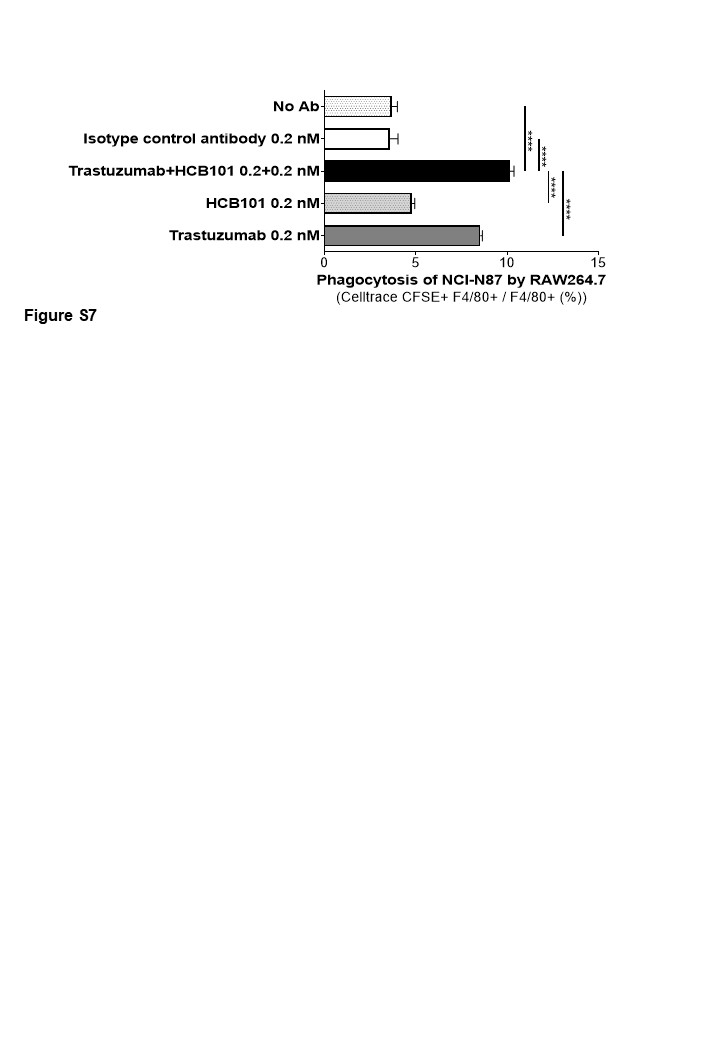

Supplement: Supplementary file 4 — Supplementary Material 4. [file 13045_2025_1742_MOESM4_ESM.jpg]

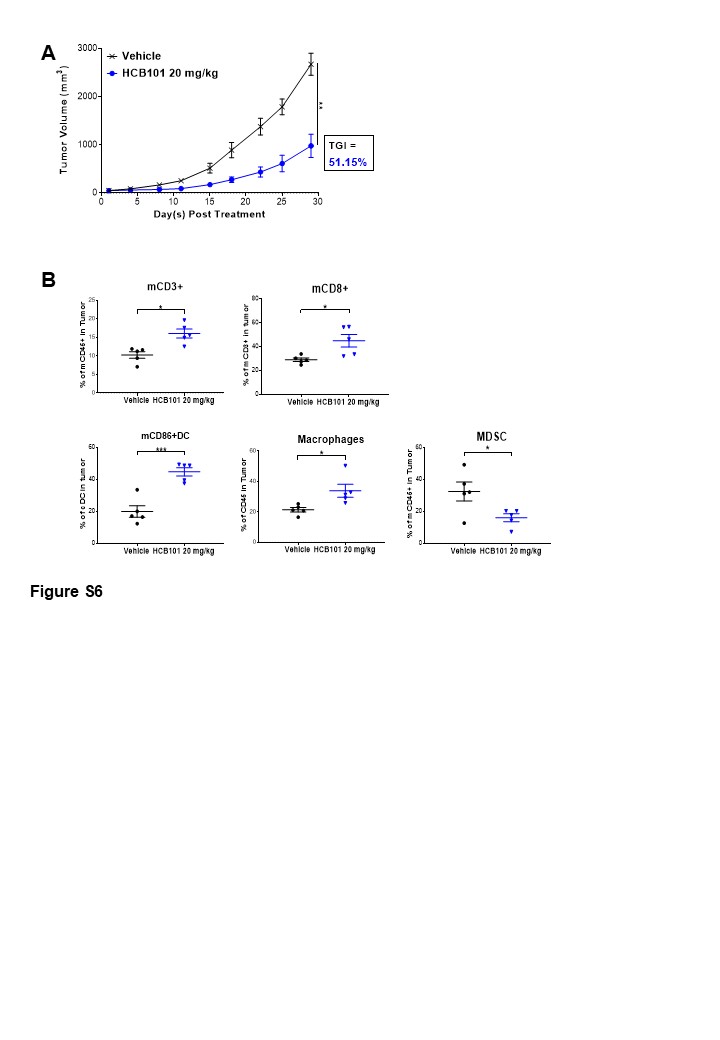

Supplement: Supplementary file 5 — Supplementary Material 5. [file 13045_2025_1742_MOESM5_ESM.jpg]

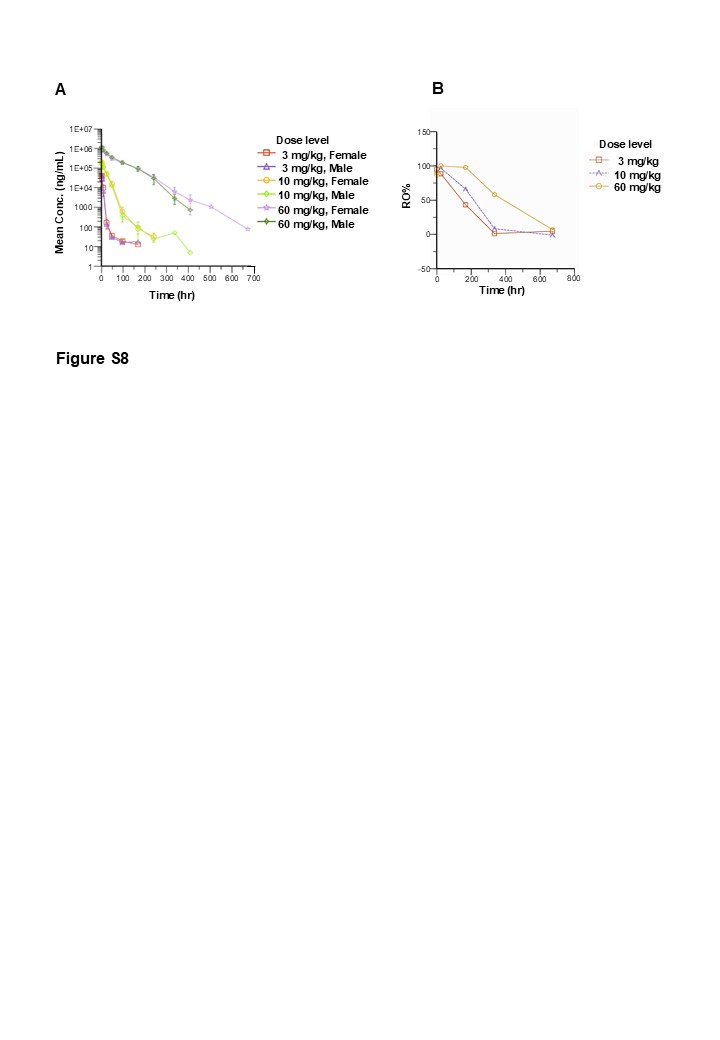

Supplement: Supplementary file 6 — Supplementary Material 6. [file 13045_2025_1742_MOESM6_ESM.jpg]

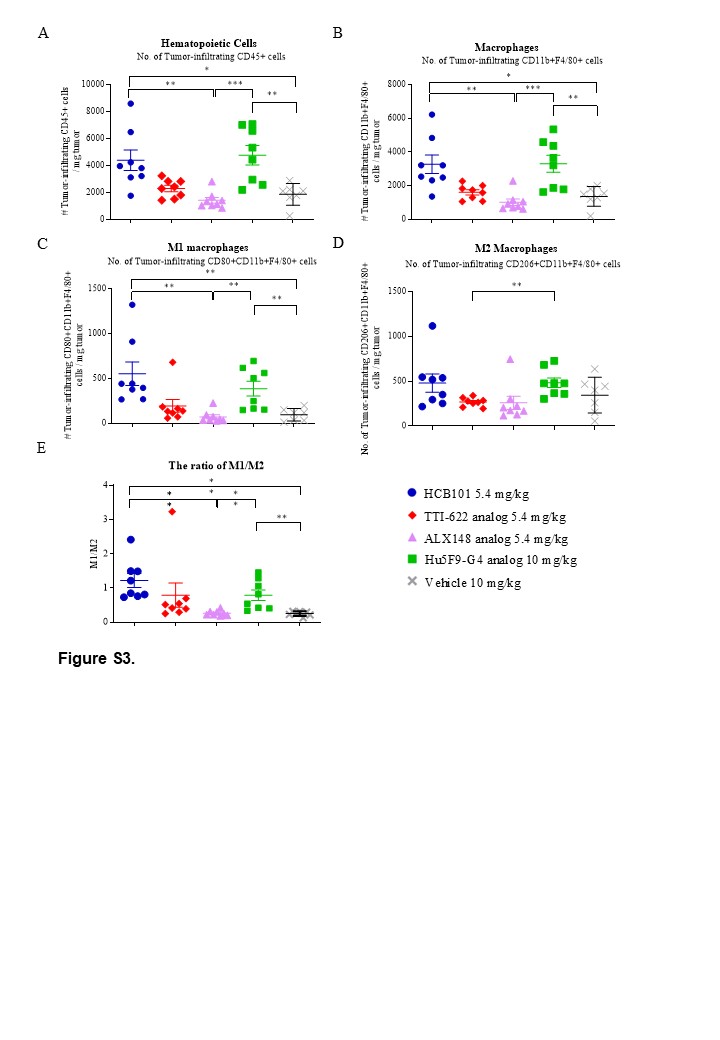

Supplement: Supplementary file 7 — Supplementary Material 7. [file 13045_2025_1742_MOESM7_ESM.jpg]

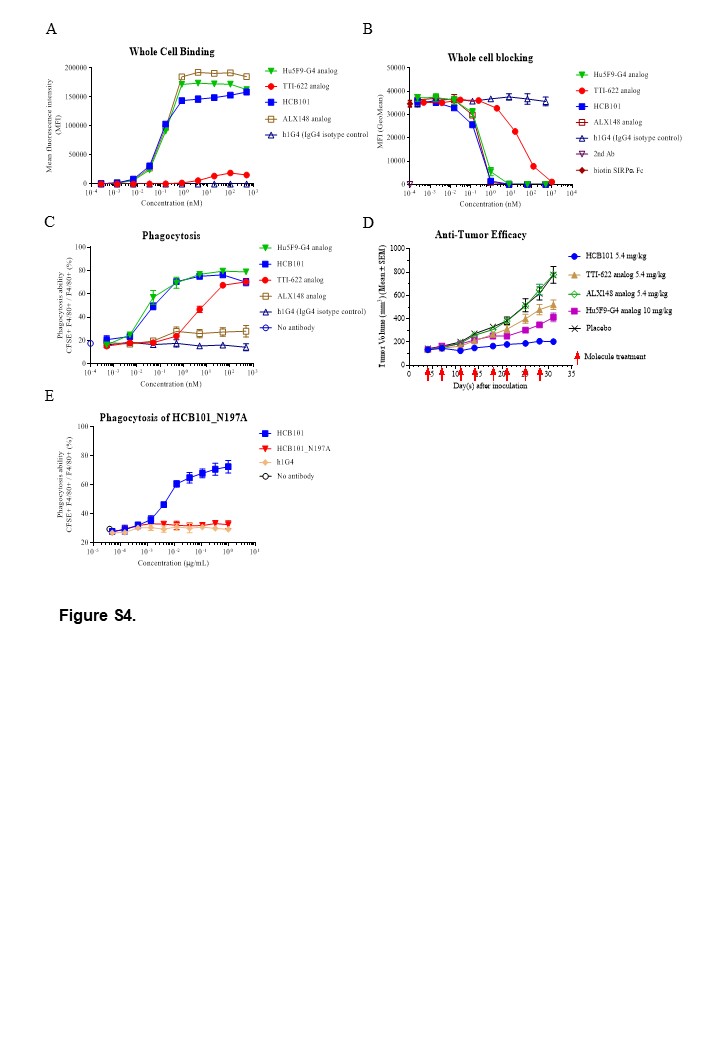

Supplement: Supplementary file 8 — Supplementary Material 8. [file 13045_2025_1742_MOESM8_ESM.jpg]

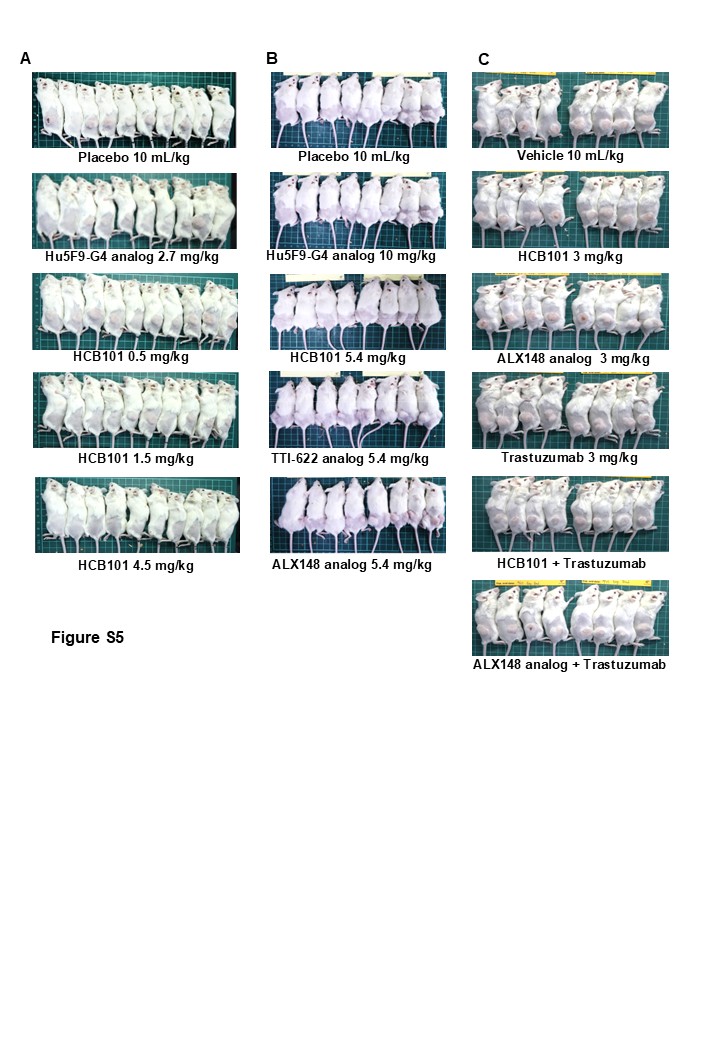

Supplement: Supplementary file 9 — Supplementary Material 9. [file 13045_2025_1742_MOESM9_ESM.jpg]
